# Unveiling the Impact of COVID-19 Vaccines: A Meta-Analysis of Survival Rates Among Patients in the United States Based on Vaccination Status

**DOI:** 10.7759/cureus.43282

**Published:** 2023-08-10

**Authors:** Anderson E Ikeokwu, Rebecca Lawrence, Egbaoghene D Osieme, Khalifa M Gidado, Cullen Guy, Oladejo Dolapo

**Affiliations:** 1 College of Medicine, Richmond Gabriel University, Kingstown, VCT; 2 School of Medicine, All Saints University Dominica, Roseau, DMA; 3 College of Medicine, Washington University of Health and Science, San Pedro, BLZ

**Keywords:** covid-19 vaccination, sars-cov-2, systematic review and meta analysis, corona virus disease 2019 (covid-19), mortality, north america, covid-19

## Abstract

The COVID-19 pandemic caused by the severe acute respiratory syndrome coronavirus 2 (SARS-CoV-2) has resulted in a significant number of cases and deaths worldwide. Vaccination is the most effective preventive measure against the disease. This study aimed to assess the mortality rates of COVID-19 patients in the United States and the effectiveness of Pfizer (Pfizer, NY, USA), Moderna (Moderna, MA, USA), and Janssen (Johnson & Johnson, NJ, USA) vaccines in preventing mortality.

A systematic review and meta-analysis were conducted following the Preferred Reporting Items for Systematic reviews and Meta-Analyses (PRISMA-2020) guidelines. Eligible studies reporting on the effectiveness of COVID-19 vaccines on patient outcomes were included. The search was performed in PubMed, Cochrane, and Google Scholar databases. The data were extracted, and risk ratios (RR) were calculated for mortality outcomes. The analysis was performed using Review Manager software, and bias assessments were conducted using the Joanna Briggs Institute (JBI) Meta-Analysis tools.

A total of seven studies with 21,618,297 COVID-19 patients were included in the meta-analysis. The odds ratio (OR) for mortality among unvaccinated patients compared to vaccinated patients was 2.46 (95% CI: 1.71-3.53), indicating that unvaccinated patients were 2.46 times more likely to die from COVID-19.

The findings of this study support the effectiveness of COVID-19 vaccination in reducing mortality among infected individuals. Unvaccinated patients had a significantly higher risk of mortality compared to vaccinated patients. Vaccination remains a crucial strategy to mitigate the severity of the disease and reduce mortality rates. Efforts should be made to address vaccine hesitancy and ensure widespread vaccine coverage.

## Introduction and background

Ever since the outbreak of the severe acute respiratory syndrome coronavirus 2 (SARS-CoV-2) in Wuhan, China, in December 2019, the world has witnessed close to 760 million confirmed cases of COVID-19 infection, and as a result close to seven million deaths as of May 2023. The WHO statistics tabulate 115 million confirmed cases and as a result 1.5 million deaths in the North American Region inclusive of the United States, Canada, and Mexico [1].

At this time, there continue to be new variants of the COVID-19 virus with increasing complications and mortality rates, thereby forcing researchers to find innovative ways to combat the disease’s lethality therapeutically and with preventative efforts. Vaccination continues to be the most accessible and safest method to prevent future reinfections and improves clinical outcomes in the case of hospitalization.

The US Coronavirus vaccine tracker states that 81% of the population has received at least one dose of the vaccine, 70% received two doses and are considered fully vaccinated whereas an additional 34% of the population has received at least one booster dose [2]. However, a sizeable portion of the public is still reluctant to get the vaccine due to concerns about safety, emergency authorization of these vaccines, mistrust in their public health systems, or misplaced complacency [3]. Therefore, we conducted a systematic review and meta-analysis to examine mortality rates of American patients infected with COVID-19 and the effectiveness of the following vaccines namely Pfizer (Pfizer, NY, USA), Moderna (Moderna, MA, USA) and Janssen (Johnson & Johnson, NJ, USA) available to the public.

## Review

Materials and methods

Study Design

To evaluate the acceptance rate of COVID-19 vaccination, a meta-analysis was performed on a collection of studies. The assessment adhered to the guidelines set forth by the Preferred Reporting Items for Systematic reviews and Meta-Analyses (PRISMA-2020) [4] to review the relevant articles. As the analysis solely utilized published data, no ethics review or approval was necessary. 

Eligibility Criteria

The criteria for inclusion, include studies that report on the effectiveness of COVID-19 vaccination on patient outcomes with COVID-19 infection. The criteria included studies after the availability of COVID-19 vaccines. All types of COVID-19 vaccines utilized in the United States were included in this review.

Population (P): We included studies with cross-sectional, case-control, cohort designs and randomized controlled trials of any age published in English from 2020 to July 10, 2022 from the United States. Case series/reports, conference papers, proceedings, articles available only in abstract form, editorial reviews, letters of communication, commentaries, systematic reviews, and qualitative studies were excluded. Articles in languages other than English or study areas not in the United States were excluded.

Intervention (I): We included all types of COVID-19 vaccines utilized in the United States in this review.

Comparison (C): We included studies that compared the patients into two groups according to their vaccination status. Individuals who received at least one dose of any COVID-19 vaccine were placed in the “vaccinated group”; individuals who did not receive any vaccine dose were placed in the “non-vaccinated group.”

Outcomes (O): Our primary outcome measures mortality due to COVID-19 infection.

Information Sources

A systematic search was conducted on April 21, 2022, utilizing three databases: PubMed, Cochrane, and Google Scholar. To identify additional relevant studies, a “snowball” search strategy was employed by examining the reference lists of publications eligible for full-text review and screening studies that cited them using Google Scholar. The database search was further updated on July 7, 2022, while the snowball and additional searches were conducted on July 8, 2022.

Search Strategy

The search was done using the generic free-text search terms developed based on the study, Patient-Intervention-Comparison-Outcome (PICO) model to define the clinical question to aid in finding clinically relevant evidence in the literature. P = “COVID-19” AND “UNITED STATES,” I = “COVID-19 VACCINE,” C = “VACCINATION STATUS” OR “VACCINATED” AND “UNVACCINATED,” O = “MORTALITY.” In order to encompass all possible and relevant studies, a broad range of search terms was utilized. All studies published between 2020 and July 10, 2022 were gathered to determine their suitability for inclusion in this study. The search was limited to full-text articles written in English. To identify any additional studies that met the inclusion criteria, the reference lists of the included citations were carefully examined.

Selection Process

**Figure 1:**
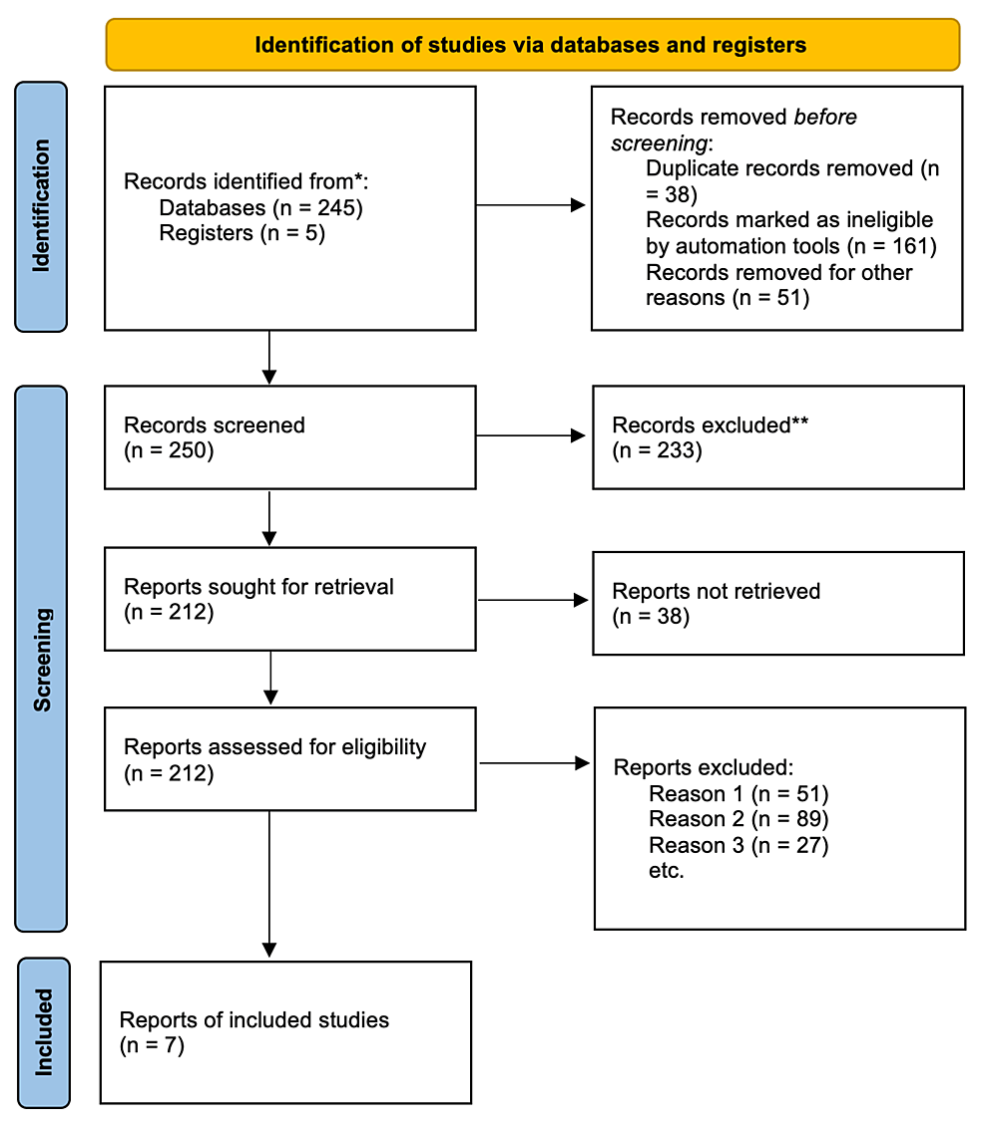
PRISMA flow diagram showcasing the inclusion criteria of studies found eligible in the meta-analysis PRISMA: Preferred Reporting Items for Systematic reviews and Meta-Analyses

Our search strategy yielded a collection of records that were exported to Rayyan Intelligent Systematic Review software (Rayyan System Inc., MA, USA) [5]. This software helps ensure data integrity by removing all duplicate articles. The initial examination of the titles and abstracts of the first 100 records was conducted independently by two researchers (AI and RL). Any disparities encountered were discussed until a consensus was reached. Subsequently, the researchers worked in pairs to evaluate the titles and abstracts of all retrieved articles. In the event of discordance, a consensus on which articles to review in the full text was achieved through discussion. If necessary, a third researcher (EO) was consulted for assistance in making the final decision. Afterward, the full-text articles were individually reviewed for inclusion by both researchers (AI and RL). Again, any differences in opinion regarding inclusion or exclusion were resolved through discussion. The search methodology employed is depicted in the PRISMA flow chart (Figure 1), which illustrates the included studies as well as those excluded along with the reasons for exclusion. The reasons for exclusion included: Reason 1: absence of comparable groups (i.e., vaccinated vs. unvaccinated), Reason 2: unavailability of the complete text, and Reason 3: lack of relevance to the research question, encompassing insufficient data on patient health outcomes.

Data Collection Process

We designed a data extraction form based, on which two review authors (AI and RL) used to extract data from eligible studies. Extracted data were compared, with any discrepancies being resolved through discussion. The data was entered into Review Manager (RevMan 2014) Version 5.3 (Cochrane, Copenhagen, Denmark) [6], double-checking this for accuracy.

Data Outcomes

The data included the first author, the year the study was published, the study's location, its design, the setting, the characteristics of the COVID-19 patients who participated in the trial and their various comorbidities, the number of doses, the sample size, the proportion, and information needed to assess the effect estimates. Death from COVID-19 infection was the specific outcome measure that was recorded for the meta-analysis. Mortality from SARS-CoV-2 was defined as death within 28 days of first testing positive for SARS-CoV-2 via PCR test [7]. The effectiveness of a COVID-19 vaccine was referred to in this study as to how well the vaccine works in preventing COVID-19 infection or reducing the severity of the disease among vaccinated individuals. It was typically measured in this study by comparing the rates of mortality between vaccinated and unvaccinated patients with COVID-19 infection.

Effect Measures

The effectiveness of COVID-19 vaccination on patient outcomes with COVID-19 infection was reported in pooled estimate proportion with a 95% confidence interval. We analyzed dichotomous outcomes by calculating the odds ratio (OR) of a patient outcome (i.e., mortality) for each study.

Synthesis Methods

The analysis was performed with the software RevMan 2014. A generic inverse variance with a random-effects model was applied to pool the proportion of the studies’ data. The heterogeneity was assessed by I2 statistic and p-value. If the p-value is < 0.05 or I2 > 50%, the assumption of homogeneity was rejected, and a random-effects model was adopted.

Study Risk of Bias Assessment

The risk of bias assessment was assessed using the Risk of Bias tool 2.0 (RoB 2.0) (Cochrane, London, United Kingdom) to assess the risk of bias for each of the included observational studies [8]. The evaluation of data quality was conducted using the Joanna Briggs Institute (J.B.I.) to critically appraise the studies included in the meta-analysis. The meta-analysis encompassed cross-sectional, case-control, cohort studies, and randomized clinical trials [9]. The risk of bias in the observational studies (case-control and cohort) was evaluated using nine criteria [9]: (1) appropriateness of the sample frame, (2) appropriateness of the sampled study participants, (3) adequacy of the sample size, (4) description of the study subjects and setting, (5) justification of sample size, power description, or variance and effect estimates, (6) valid methods for identifying the condition, (7) standardized and reliable measurement of the condition, (8) appropriateness of statistical analysis, and (9) adequacy of the response rate. The risk assessment criteria were categorized as “yes,” “no,” “unclear,” or “not available.” A score of one (1) was assigned for “yes” responses, while a score of zero (0) was given for the remaining categories. The risk of bias was considered low when the total score exceeded 70%, moderate when it ranged from 50% to 69%, and high when it fell between 0% and 49% [9]. Two authors independently performed the bias assessments.

Rating Evidence of Quality

We used the Grading of Recommendations Assessment, Development, and Evaluation (GRADE) approach to rate the quality of evidence the clinical outcome (mortality from COVID-19 infection), as high, moderate, low or very low [10]. The assessment included judgments addressing the risk of bias, imprecision, inconsistency, indirectness, and publication bias low [10]. If there were serious concerns in any of these domains (for instance, in risk of bias), we rated the quality of the evidence low [10]. The GRADEpro Guideline Development Tool (GDT) software (McMaster University, ON, Canada and Evidence Prime, Kraków, Poland) was utilized to rate the quality of evidence [11].

Results

We identified 250 published papers in database searching. Across all of these papers, there were 21,618,297 COVID-19 patients. A total of 240 articles from PubMed and 10 from the Cochrane database were identified from the initial search. Following duplicate removal, 167 articles were excluded in accordance with the inclusion and exclusion criteria. We finally selected seven articles for the meta-analysis (Table 1).

**Table 1:** Sample size of selected studies and their characteristics MMWR: Morbidity and Mortality Weekly Report, CDC: Centers for Disease Control and Prevention, HR: Hospitalization rate, MV: Mechanical ventilation, CC: COVID-19 cases, ICU: Intensive care unit, MR: Mortality rate, VT: Tidal volume

Author (Year)	Study Area	Study Type Design	Journal name	Total number of patients	Outcomes analyzed	Vaccine type
Naleway (2021) [12]	USA	Retrospective Cohort	MMWR (CDC)	482,464	HR, MV, CC, ICU, MR	Pfizer, Moderna, Janssen (J&J)
Johnson (2022) [13]	USA	Retrospective Cohort	MMWR (CDC)	9,678,557	CC, MR	Unspecified
Danza (2022) [14]	USA	Cross-sectional	MMWR (CDC)	422,966	HR, MV, ICU, MR,	Pfizer, Moderna, Janssen (J&J)
Olson (2022) [15]	USA	Case-control	The New England Journal of Medicine	1,222	HR, VT, ICU, MR	Pfizer
Griffin (2021) [16]	USA	Cross-sectional	MMWR (CDC)	43,127	HR, MV, CC, ICU, MR	Pfizer, Moderna, Janssen (J&J)
Tenforde (2022) [17]	USA	Case-control	Jama Network	1,983	HR, MR,	Pfizer, Moderna
Xu (2021) [18]	USA	Retrospective Cohort	MMWR (CDC)	10,987,919	MR	Pfizer, Moderna, Janssen (J&J)

In a review examining the effectiveness of COVID-19 vaccination on patient outcomes with COVID-19 infection, the authors included a table presenting for each included study the citation, study design, country, sample size, median age, male: female and ethnicity distribution of vaccinated and unvaccinated patients, patient comorbidities, and type of COVID-19 vaccine used of various studies have been elaborated in Tables 2-6. In this analysis, mortality in various studies is considered a clinical outcome in patients with COVID-19 infections. 

**Table 2:** Summary of demographics data N: Number of patients vaccinated/unvaccinated, %: Percentage of patients vaccinated/unvaccinated in relation to the total number of patients

Author (Year)	Total number of patients	N (%) Vaccinated	N (%) Unvaccinated	N (%) Female	N (%) Male	Age Range of patients
Naleway (2021) [12]	482,464	344,848 (71.5)	137,616 (28.5)	251,552 (52.1)	230,552 (47.8)	18-75
Johnson (2022) [13]	9,678,557	2,866,517 (29.6)	6,812,040 (70.4)	-	-	18-65+
Danza (2022) [14]	422,966	281,038 (66.4)	141,928 (33.6)	224,173 (53)	184,134 (43.5)	18-80+
Olson (2022) [15]	1,222	345 (28.2)	868 (71.8)	-	-	12-18
Griffin (2021) [16]	43,127	12,326 (28.6)	30,801 (71.4)	21,743 (50.4)	20,425 (47.4)	16-80+
Tenforde (2022) [17]	1,983	314 (15.8)	1,669 (84.2)	969 (48.9)	1,014 (51.1)	18-65+
Xu (2021) [18]	10,987,919	6,398,361 (58.2)	4,589,557 (41.8)	5,946,533 (54.1)	5,041,385 (45.9)	12-85+

**Table 3:** Summary of demographics data for vaccinated patients N: Number of patients vaccinated/unvaccinated, %: Percentage of patients vaccinated/unvaccinated in relation to the total number of patients

Author (Year)	N (%) Vaccinated	N (%) Female	N (%) Male	Mean/Median age	N (%) White	N (%) Asian	N (%) Black	N (%) Hispanic	N (%) Native American	N (%) Native Hawaiian/pacific islander	N (%) Multiple races/others/unknown
Naleway (2021) [12]	344,848	187,711 (54.5)	156,960 (45.5)	50	242,110 (70.2)	22,828 (6.6)	8,224 (2.4)	-	1,2880 (0.4)	1,931 (0.6)	68,475 (19.9)
Johnson (2022) [13]	2,866,517	-		-	-	-	-	-	-	-	-
Danza (2022) [14]	281,038	154,791 (55.1)	117,971 (42)	36	46,612 (16.6)	26,384 (9.4)	15,991 (5.7)	-	530 (0.2)	2,348 (0.8)	40,538 (14.4)
Olson (2022) [15]	345	-	-	16	143 (41.4)	-	68 (19.7)	94 (27.2)	-	-	49 (14.2)
Griffin (2021) [16]	12,326	6,271 (50.9)	5,908 (47.9)	36	3,718 (30.2)	1,009 (8.2)	819 (6.6)	3,961 (32.1)	19 (0.2)	49 (0.4)	2,447 (19.9)
Tenforde (2021) [17]	314	138 (44)	176 (56)	67	201 (64)	-	55 (17.5)	44 (14)	-	-	14 (4.5)
Xu (2021) [18]	6,398,361	3,448,362 (53.9)	2,949,999 (46.1)	-	2,778,730 (43.4)	633,212 (10)	341,189 (5.3)	1,409,187 (22)	-	-	880,523 (13.8)

**Table 4:** Summary of demographics data for unvaccinated patients N: Number of patients vaccinated/unvaccinated, %: Percentage of patients vaccinated/unvaccinated in relation to the total number of patients

Author (Year)	N (%) Unvaccinated	N (%) Female	N (%) Male	Mean/Median age	N (%) White	N (%) Asian	N (%) Black	N (%) Hispanic	N (%) Native American	N (%) Native Hawaiian/pacific islander	N (%) Multiple races/others/unknown
Naleway (2021) [12]	137,616	63,841 (46.4)	73,592 (53.5)	37	83,474 (60.7)	3,930 (2.9)	4,851 (3.5)	-	588 (0.4)	1,021 (0.7)	43,752 (31.8)
Johnson (2022) [13]	6,812,040	-	-	-	-	-	-	-	-	-	-
Danza (2022) [14]	141,928	69,382 (48.9)	66,163 (46.6)	35	20,529 (14.5)	7,451 (5.2)	12,319 (8.7)	-	342 (0.2)	1,429 (1)	19,214 (1305)
Olson (2022) [15]	868	-	-	15	358 (41.2)	-	197 (22.7)	191 (22)	-	-	122 (14)
Griffin (2021) [16]	30,801	15,472 (50.2)	14,517 (47.1)	32	5,620 (18.2)	961 (3.1)	4,755 (15.4)	10,183 (33.1)	51 (0.2)	161 (0.5)	8,551 (27.8)
Tenforde (2021) [17]	1,669	831 (49.8)	838 (50.2)	53	717 (43)	-	453 (27.1)	381 (22.8)	-	-	118 (7.1)
Xu (2021) [18]	4,589,557	2,498,171 (54.4)	2,091,386 (45.6)	-	1,982,834 (43.2)	633,212 (13.8)	262,766 (5.7)	1,201,784 (26.2)	-	-	508,961 (11.1)

**Table 5:** Summary of patient comorbidities for vaccinated patients N: Number of patients vaccinated/unvaccinated, %: Percentage of patients vaccinated/unvaccinated in relation to the total number of patient

Author (Year)	Total number vaccinated	N (%) chronic kidney disease	N (%) Diabetes	N (%) Chronic lung disease	N (%) cardiovascular disease	N (%) Immunodeficiency disorder	N (%) Neuromuscular/Neurological disorder
Naleway (2021) [12]	344,848	32 (0.009)	24 (0.007)	24 (0.007)	-	-	-
Johnson (2022) [13]	2,866,517	-	-	-	-	-	10 (0.0003)
Danza (2022) [14]	281,038	-	-	-	-	-	-
Olson (2022) [15]	345	-	28 (8.1)	81 (23.5)	27 (7.8)	-	-
Griffin (2021) [16]	12,326	-	-	-	-	-	-
Tenforde (2021) [17]	314	-	112 (35.7)	100 (31.8)	236 (75.2)	128 (40.8)	-
Xu (2021) [18]	6,398,361	-	-	-	-	-	-

**Table 6:** Summary of patient comorbidities for unvaccinated patients N: Number of patients vaccinated/unvaccinated, %: Percentage of patients vaccinated/unvaccinated in relation to the total number of patients

Author (Year)	N (%) Unvaccinated	N (%) chronic kidney disease	N (%) Diabetes	N (%) Chronic lung disease	N (%) cardiovascular disease	N (%) Immunodeficiency disorder	N (%) Neuromuscular/Neurological disorder
Naleway (2021) [12]	137,616	37 (0.03)	98 (0.07)	22 (0.02)	-	-	-
Johnson (2022) [13]	6,812,040	-	-	-	-	-	15 (0.0002)
Danza (2022) [14]	141,928	-	-	-	-	-	-
Olson (2022) [15]	868	-	72 (8.3)	241 (27.8)	69 (8)	-	-
Griffin (2021) [16]	30,801	-	-	-	-	-	-
Tenforde (2021) [17]	1,669	-	425 (25.5)	327 (19.6)	814 (48.8)	191 (11.4)	-
Xu (2021) [18]	4,589,557	-	-	-	-	-	-

Risk of Bias Assessment

**Figure 2:**
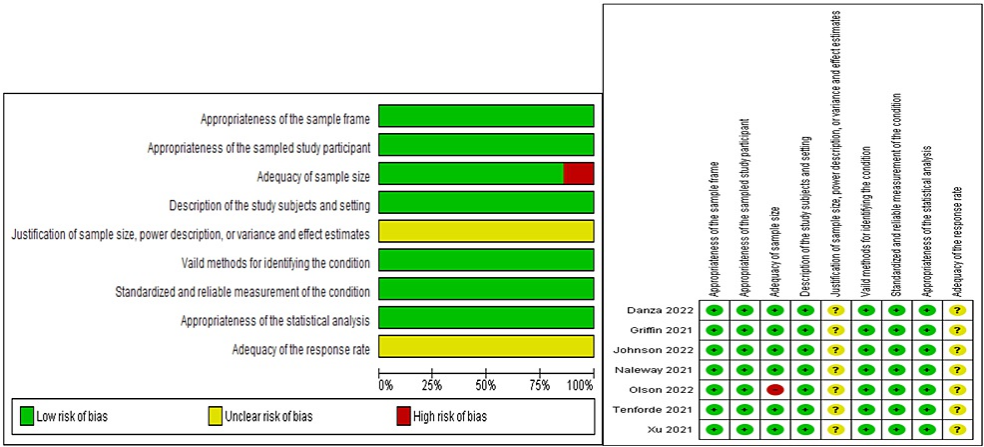
Risk bias assessment

In terms of overall risk bias, the risk of bias was low. There were concerns about the uncertain risk of bias in two out of the nine criteria for all seven studies included. These two criteria were justification of sample size and adequacy of response rate. All of the studies did not report enough data to justify the sample size or assess the adequacy of the response rate. Regarding the adequacy of sample size, one study [15] was at high risk of bias. A summary of these assessments is provided in Figure 2.

Analysis of Mortality From COVID-19 by Vaccination Status

**Figure 3:**
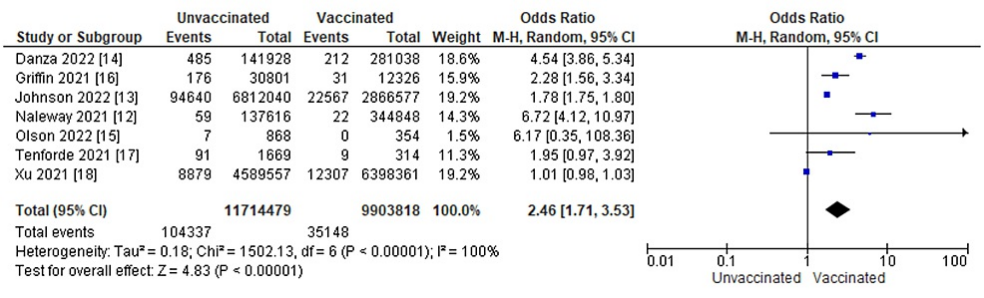
Mortality from COVID-19 infection by vaccination status The figure displays for each study included in the meta-analysis a summary of statistics (number of events and sample size) for the unvaccinated and vaccinated groups, the Odds Ratio (OR) and its 95% Confidence Interval (CI), heterogeneity, and test for overall effect for the dichotomous outcome mortality from COVID-19 Infection [12-18].

The patient outcomes in COVID-19 patients were compared between those who received the COVID-19 vaccine and those who did not. In the seven studies analyzed, a total of 139,485 patients were reported to have died from COVID-19 infection. The OR of COVID-19 mortality between patients with COVID-19 vaccination versus patient without COVID-19 vaccination was 2.46 with a 95% CI ranging from 1.71 to 3.53. The result was statistically significant which indicates that unvaccinated patients with COVID-19 infection are 2.46 times more likely to die from COVID-19 infection compared to those who are vaccinated with COVID-19 infection (p < 0.0001). A heterogeneity test was done with results of I2 = 100%, p = <0.00001 (Figure 3).

GRADE Summary of Findings

**Figure 4:**
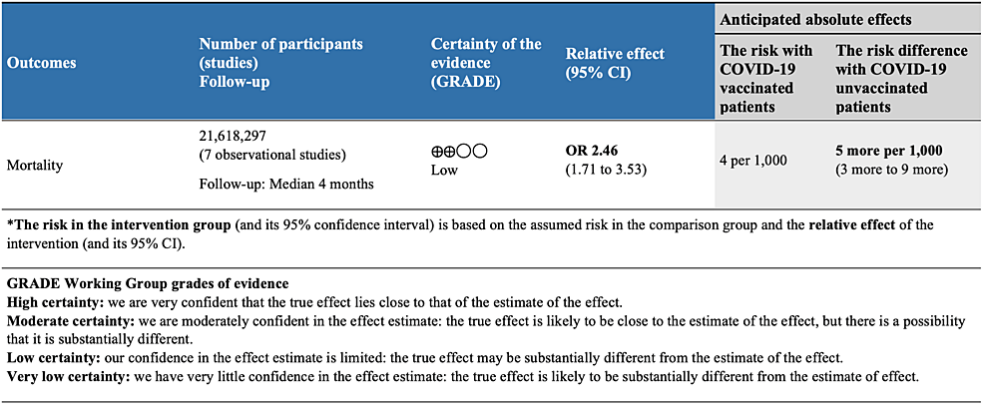
GRADE summary of findings GRADE: Grading of Recommendations Assessment, Development, and Evaluation

Evidence for mortality from COVID-19 infection by vaccination status was available from seven observational studies included a total of 21,618,297 patients (Figure 4). After rating down one level for study design, we considered the evidence to be low-quality for an observational study design. These observational studies suggest that COVID-19 vaccination may substantially reduce mortality (OR 2.46, 95% CI 1.71 to 3.53; low-quality evidence) (Figure 3). 

Discussion 

This study evaluated the effectiveness of COVID-19 vaccination on patients with COVID-19 infection in the United States. In this evaluation, several outcomes were analyzed among individuals within the age range of 12-95 years using the following study design types: retrospective cohort, cross-sectional, randomized control trial and case-control study. Analysis of these studies reveals that COVID-19 vaccination confers a certain level of protection against poor outcomes in COVID-19-infected individuals.

The pandemic COVID-19 has had a global impact on mortality and morbidity. Vaccination has been linked to a considerable decrease in the number of symptomatic COVID-19 infections in adults as well as improved protection against severe disease [19-21]. Patients who were fully vaccinated were less likely to develop critical illness and require intensive care and were thus discharged faster [22-25]. Inadequate immunity in unvaccinated patients, combined with the growing prevalence of the delta variation, resulted in greater illness and fatality rates [26,27]. As the severity of the disease worsens, mortality rises dramatically [15,16]. Comorbidity risk influences both illness progression and mortality [28,29]. In this study, vaccinated individuals had considerably reduced mortality than unvaccinated patients.

In line with the majority of the articles used for this meta-analysis study, we noted that unvaccinated patients infected with COVID-19 are 2.46 times more likely to die from the COVID-19 infection compared to those that are vaccinated but infected with the virus. Our study is also in support of previous studies such as Tenforde, where it was noted that among patients hospitalized with COVID-19, the outcomes of death or invasive mechanical ventilation were associated with a lower likelihood when fully or partially vaccinated [17]. Moreso, in tandem with the study of Xu et al., it was noted that in a cohort of 6.4 million COVID-19 vaccines and 4.6 million demographically similar unvaccinated persons, recipients of the Pfizer-BioNTech, Moderna, or Jensen vaccines had lower non-COVID-19 mortality risks compared to the unvaccinated comparison group [18]. They also noted that there is no increased risk for mortality among COVID-19 vaccine recipients and this finding reinforces the safety profile of currently approved COVID-19 vaccines in the United States.

The risk of mortality among patients with COVID-19 infection is influenced by their sociodemographic characteristics, with an increased risk observed among those who are unvaccinated. In a recent study that compared mortality, recovery rates, and disease severity between men and women using a random-effects meta-analysis [30], the analysis found that male patients have a higher risk of mortality and a lower chance of recovery compared to female patients. Additionally, male patients were more likely to present with a severe form of COVID-19. The male-to-female ratio for cases was 1:0.9 [29]. This study however showed that there was no striking difference between males and females regarding disease susceptibility. The study however showed that the course of COVID-19 is more severe in men, but the vaccine may improve the prognosis in men, as fully vaccinated patients had a significantly higher mean age than unvaccinated and under-vaccinated patients [30].

Men may be more susceptible to COVID-19 due to differences in innate immunity, steroid hormones, and sex chromosomal characteristics [30,31]. Females may be at an advantage due to increased TLR7 and CD4+ cell expression, which ensures better elimination of viruses [31]. Male patients infected with COVID-19 have a 61% greater likelihood of dying from the infection than their female counterparts [31]. Cytokine storms have been observed as more frequently occurring in men, leading to multi-organ failure and death. Men had poorer rates of recovery and have longer viral RNA shedding for SARS-CoV-2, implying a slower recovery. Hormonal variables may also have an impact on disease phenotype and severity [28,32,33].

Age is a prognostic factor in determining the risk of mortality in patients with COVID-19 infections. This study included 486 patients with COVID-19, with 54.3% of them being unvaccinated. The median age was 53 years for unvaccinated and partially vaccinated patients and 62 years for fully vaccinated patients [28]. Critical illness was more prevalent in unvaccinated or partially vaccinated patients, and older age, higher disease severity, higher comorbidity index, and not being fully vaccinated were factors associated with higher mortality. The study highlights the importance of vaccination in reducing the severity of the disease and mortality, particularly in older patients with comorbidities [28].

It has been suggested that racial health disparities have contributed to an increased risk of mortality from COVID-19 infection. A systematic review and meta-analysis by Pal et al. showed that Native American men had the highest mortality risk [34]. Studies have also reported higher mortality rates among Black people, but this study found a similar risk of mortality among Black men compared to White men. These discrepancies between studies also may be due to different timing of sampling and trends in COVID-19 infection among different racial identity groups [35].

In contrast, another study examined the characteristics and outcomes of COVID-19 patients in California, Oregon, and Washington across different races/ethnicities [36]. The study found that Hispanic patients were disproportionately affected and had increased odds of hospital mortality. Other minority races/ethnicities were not significantly associated with increased mortality [36].

Limitations

There are several key limitations to our study that should be mentioned, such as the fact that the risks of COVID-19 infection are not the same for everyone, therefore the chance of exposure may influence the likelihood of COVID-19 vaccine acceptance and coverage. Finally, we were unable to independently assess the preventative impact of single doses against double and booster doses, as well as independently assess the effectiveness of the specific vaccines approved in the United States against distinct virus strains and clinical outcomes. Possible explanations include a lack of consistency in vaccine schedules and availability in the United States.

## Conclusions

The meta-analysis study reviewed here provides evidence that COVID-19 vaccination confers a certain level of protection against poor outcomes in individuals infected with the virus. The study found that unvaccinated patients with COVID-19 are 2.46 times more likely to die from the infection compared to those that are vaccinated. Additionally, the study highlights the importance of vaccination in reducing the severity of the disease and mortality, particularly in older patients with comorbidities. Based on the findings of this study, it is recommended that individuals receive the COVID-19 vaccine as a means of protecting themselves against severe disease and mortality associated with COVID-19 infection.

Governments and health organizations should continue to encourage and facilitate vaccination efforts, particularly amongst high-risk populations such as the elderly and those with underlying health conditions. Efforts should also be made to address health disparities in access to and uptake of COVID-19 vaccines to ensure equitable distribution and protection for all populations. The study also emphasizes the need for data collection, improving access to testing, and the need for active intervention earlier in the disease course in addition to culturally appropriate public health messaging. The report also emphasizes the necessity of racial equity in vaccination distribution as well as the need for diversity in clinical trials to guarantee the safety and effectiveness of vaccines and therapies.
